# Behavioral and neurogenomic transcriptome changes in wild-derived zebrafish with fluoxetine treatment

**DOI:** 10.1186/1471-2164-14-348

**Published:** 2013-05-24

**Authors:** Ryan Y Wong, Sarah E Oxendine, John Godwin

**Affiliations:** 1 Department of Biology, North Carolina State University, Box 7617, Raleigh, NC 27695-7617, USA; 2 WM Keck Center for Behavioral Biology, North Carolina State University, Box 7617, Raleigh, NC 27695-7617, USA

**Keywords:** Fluoxetine, Anxiety, Stress, SSRI, Genomic, Brain, RNA-sequencing, Zebrafish, Serotonin, GABA

## Abstract

**Background:**

Stress and anxiety-related behaviors are seen in many organisms. Studies have shown that in humans and other animals, treatment with selective serotonin reuptake inhibitors (e.g. fluoxetine) can reduce anxiety and anxiety-related behaviors. The efficacies and side effects, however, can vary between individuals. Fluoxetine can modulate anxiety in a stereospecific manner or with equal efficacy regardless of stereoisomer depending on the mechanism of action (e.g. serotonergic or GABAergic effects). Zebrafish are an emerging and valuable translational model for understanding human health related issues such as anxiety. In this study we present data showing the behavioral and whole brain transcriptome changes with fluoxetine treatment in wild-derived zebrafish and suggest additional molecular mechanisms of this widely-prescribed drug.

**Results:**

We used automated behavioral analyses to assess the effects of racemic and stereoisomeric fluoxetine on male wild-derived zebrafish. Both racemic and the individual isomers of fluoxetine reduced anxiety-related behaviors relative to controls and we did not observe stereospecific fluoxetine effects. Using RNA-sequencing of the whole brain, we identified 411 genes showing differential expression with racemic fluoxetine treatment. Several neuropeptides (neuropeptide Y, isotocin, urocortin 3, prolactin) showed consistent expression patterns with the alleviation of stress and anxiety when anxiety-related behavior was reduced with fluoxetine treatment. With gene ontology and KEGG pathway analyses, we identified lipid and amino acid metabolic processes, and steroid biosynthesis among other terms to be over-enriched.

**Conclusion:**

Our results demonstrate that fluoxetine reduces anxiety-related behaviors in wild-derived zebrafish and alters their neurogenomic state. We identify two biological processes, lipid and amino acid metabolic synthesis that characterize differences in the fluoxetine treated fish. Fluoxetine may be acting on several different molecular pathways to reduce anxiety-related behaviors in wild-derived zebrafish. This study provides data that could help identify common molecular mechanisms of fluoxetine action across animal taxa.

## Background

Many organisms experience stress from natural sources such as territory defense, food competition, resource acquisition and exploration. In humans persistent extreme responses to stressful and anxiety-inducing situations or the inability to cope with such scenarios is a characteristic of an anxiety disorder [1]. Pharmacological studies indicate that anxiety and anxiety-related behaviors are associated with dysregulation of at least one of several major neurotransmitter systems in the brain: serotonergic (serotonin), GABAergic (gamma-aminobutyric acid), catecholaminergic (noradrenaline, dopamine) and glutamatergic (glutmate) systems [2,3]. Efficacies of various anxiolytic compounds, however, vary between individuals (both human and animal models) [4]. The extent to which this can be attributed to the nonselective or pleiotropic molecular aspects of the anxiolytic is unknown.

Selective serotonin reuptake inhibitors (SSRIs) are typically the first-line drugs prescribed for the alleviation of anxiety disorders in humans [5]. SSRI compounds (e.g., fluoxetine, citalopram, sertraline) block the serotonin transporter thus allowing for serotonin levels to remain high for longer periods of time in the synapse. Fluoxetine (tradename: Prozac) is a racemic mixture where both the R and S isomers effectively inhibit the serotonin transporter [6-10] and has also been demonstrated to have anxiolytic effects in rodents and fish [11,12]. While SSRIs are known to modulate serotonin levels, it has been shown that they also modulate production of neurosteroids (e.g. allopregnanolone) that act as modulators of GABA receptors [13-16]. In fluoxetine, S-fluoxetine induces greater allopregnanolone production in rodent brains than the R isomer [6,14]. Increasing allopregnanolone levels in rodents has been associated with decreases in aggression and anxiety [6,15,17-23]. In addition, SSRIs also affect various neuropeptide levels (e.g., neuropeptide Y, oxytocin, arginine vasopressin), which independently can modulate stress and anxiety levels [24]. Our laboratory has shown fluoxetine can affect expression of neuropeptide genes in teleosts [25]. Hence SSRIs appear to affect many systems and understanding the full extent of changes in molecular mechanisms underlying fluoxetine mediated reduction in anxiety and stress is needed.

To understand the mechanisms underlying stress and anxiety, animal models used are highly inbred (i.e., domesticated) or selectively bred to exhibit extreme behavioral and physiological responses to stressful and anxiety-inducing contexts [2,11,26-29]. Although rodents have been primarily used as animal models for anxiety [2,26], zebrafish (*Danio rerio*) is an emerging system and valuable translational model for understanding stress and anxiety-related behaviors [30-35]. Similar to rodents, highly inbred lines of zebrafish show differences in baseline levels of stress and anxiety as well as responses to anxiolytics [2,4,11,12,36,37]. Zebrafish share similar neurochemistry, and behavioral and physiological responses to both anxiolytic and anxiogenic drugs as mammals [11,30,31]. Several neurotransmitter systems thought to be modulated in stress and anxiety (e.g., catecholamines, serotonin) in mammals are also described in zebrafish [32,38,39]. Zebrafish possess genes that encode serotonin transporters homologous to those of tetrapods, and the transporters have been shown to transport serotonin across cell membranes and the activity can be reduced with a SSRI *in vitro*[40]. Further, SSRI binding kinetics to the serotonin transporter in teleosts are similar in magnitude to rats [41]. Adult domesticated zebrafish also show reduced anxiety-related behaviors and cortisol levels when treated with fluoxetine [11]. Larval zebrafish treated with fluoxetine show changes in molecular pathways associated with stress response [42], but very little is known of the gene expression changes in adults. Hence, in addition to the zebrafish system’s amenability for genetic manipulations and high throughput drug screening [43-45], the zebrafish represents a powerful model to explore molecular mechanisms of stress and anxiety-related behaviors.

In this study we assess the effects of chronic fluoxetine treatment on behavior and the neural transcriptome of adult wild-derived male zebrafish (High Stationary Behavior line, HSB, [29]). The HSB line shows consistently high levels of stress and anxiety-related behaviors across multiple assays [29]. Given documented stereospecific fluoxetine effects on aggressive behavior and anxiety-mediated neurochemical pathways in rodents [14,21], we test whether anxiety-related behaviors can be modulated stereospecifically in zebrafish by administering racemic, R-fluoxetine, and S-fluoxetine. To analyze the underlying mechanism of fluoxetine-induced anxiolytic effects of behavior, we use RNA-sequencing and test for whole-brain differential expression of protein-coding genes between racemic fluoxetine treatment and control. Through both behavioral and gene expression analyses, we show that fluoxetine alters both behavior and neurogenomic profiles.

## Methods

### Pharmacological manipulation

Male zebrafish from our selectively bred High Stationary Behavior line (see [29] for line characteristics) were subjected to one of four treatments: a racemic mixture of fluoxetine (n = 30), R-fluoxetine (n = 12), S-fluoxetine (n = 12), or our solvent (water) as a control (n = 42). Studies in other teleosts have shown that waterborne fluoxetine enters the brain and has an approximate half life of nine and three days in the fish and water containing fish, respectively [46-48]. Testing of the racemic and stereoisomer treatment groups were separated by two months.

The first set of experiments involved treating zebrafish with a racemic fluoxetine mixture or the vehicle control. Treatment duration and racemic fluoxetine concentration follow a previously established protocol [11]. Briefly, fish were chronically treated with 100 μg/L of racemic fluoxetine (Sigma). As it has been previously demonstrated that two weeks of exposure to racemic fluoxetine alters behavior and cortisol levels [11], we assessed whether behavioral changes can be detected on a shorter time scale and thus behaviorally tested fish (n = 12, see below) treated for one week. However, to be consistent with methods in the literature and to make our gene expression results comparable with previous studies, we treated a different set of fish (n = 18) with racemic fluoxetine for two weeks. During treatment, the fish were housed in 2 L tanks in groups of six with constant aeration and fed *ad libitum* daily with commercial food (Tetramin). Every two days, we replaced all of the holding water of the fish with fresh system water (water used for normal housing) containing 100 μg/L racemic fluoxetine. Control fish (n = 42) underwent identical procedures with the exception that the solvent (water) was added to the tanks. For the second set of experiments we administered isomers of fluoxetine and controls to a different set of fish. Fish treated with a stereoisomer of fluoxetine were handled exactly the same way as above, but were treated at a concentration of 33 μg/L for two weeks. As no previous study has administered isomers of fluoxetine in teleosts, our goal was to identify a dose that would maximize the opportunity to observe differences in the behavioral effects of the two isomers in the same manner conducted in rodents [21]. We determined that 33 μg/L is a biologically relevant dose through pilot dose-response analysis where we treated individuals for two weeks as described above but at four different concentrations (Additional file 1: Figure S1). We experienced minimal deaths and all fish consumed food during the treatment period, which suggests all were in good health (final sample size: racemic fluoxetine (n = 28), R-fluoxetine (n = 11), S-fluoxetine (n = 11), control (n= 37)).

A subset of fish were treated with racemic fluoxetine (n=18) or water (control, n = 18) but did not undergo behavioral testing. These fish were decapitated and brains removed and stored in RNAlater (Ambion) until processing for either RNA-sequencing or qRT-PCR (see below). The remaining racemic fluoxetine treated fish, those treated with a fluoxetine isomer and control fish were behaviorally tested (see below). All fish used in this study were sexually mature, 7 – 11 months old, and five generations removed from the wild with the exception of a subset of males used in the qRT-PCR experiment, which were sixth generation.

### RNA-sequencing analysis

Using RNA-sequencing, we quantified whole-brain transcriptome levels in male zebrafish (five generations removed from the wild) treated with a racemic mixture of fluoxetine (n = 9) and control animals (n =10). We extracted RNA using RNeasy Plus Mini Kit (Qiagen) according to the manufacturer’s protocol. Since we wanted to assess a general effect of fluoxetine on gene expression, we pooled one microgram of total RNA from each individual in a treatment (e.g. one pooled sample for fluoxetine-treated and control fish). RNA quality was assessed with an Agilent 2100 Bioanalyzer (Agilent) and all samples had RNA integrity numbers (RIN) above 8.5. RNA samples were then submitted to the Genomic Sciences Laboratory at North Carolina State University, for cDNA library preparation (TruSeq RNA Sample Prep v2, Illumina) and 72 bp single-end RNA-sequencing (Illumina GAIIx). Following a balanced block design [49], both samples were multiplexed and run across three lanes. During analysis we combined reads across all lanes that passed default quality control filters (Illumina), which generated approximately 30.6 million reads for each of the control and fluoxetine-treated groups. Reads were aligned to the *Danio rerio* genome (assembly Zv9 [35], release 68) using GSNAP [50] with default parameters. We used Cufflinks (ver 2.0.1, [51-53]) to assess differential expression of protein-coding genes between control and fluoxetine-treated fish employing the program’s multi-read and fragment bias correction. We used gProfiler [54,55] to determine significantly over-enriched Gene Ontology (GO) terms and Kyoto Encyclopedia of Genes and Genomes (KEGG) pathways. In Cufflinks and gProfiler we utilized the default false discovery rate (FDR) corrections. Statistical significance was assessed when p_FDR-corrected_ < 0.05.

### Real-time PCR validation analysis

We assessed the expression of seven genes (*prl2, npy, oxtl, slc6a4a, slc6a4b, slc6a11, ucn3l*) associated with anxiety-like behaviors in other organisms, two genes showing more than two fold differences (*isg15, nrbf2*) in expression between treatments, and one housekeeping gene (*ef1a*) using quantitative reverse transcriptase PCR (qRT-PCR). Of these genes *prl2*, *npy*, *oxtl*, *slc6a11*, *ucn3l*, *isg15*, and *nrbf2* were differentially regulated with RNA-sequencing (see below). We used 12 fish treated with racemic fluoxetine and 12 control fish. Six fish in each treatment group were independent from those used in RNA-seq analysis (six generations removed from the wild). Total RNA from all brains was extracted following the same protocol used for RNA-sequencing preparations (see above). Genomic DNA was removed by column filtration according to manufacturer’s protocol (RNeasy Plus Mini kit, Qiagen). We subsequently quantified the RNA using Quant-iT RiboGreen (Invitrogen). Eight microliters of total RNA from each individual was reverse transcribed into cDNA using SuperScript III First-Strand Synthesis System for qRT-PCR (Invitrogen) according to modified manufacturer’s protocol where the sample was primed with both random hexamers and oligo(dT)_20_ primers. The cDNA was then purified using Amicon Ultracentrifugal filters (Millipore) according to the manufacturer’s protocol [56-58]. The resulting purified samples were topped off to 100 μl total volume with Nuclease-Free water (Ambion).

The qRT-PCR was run on an ABI 7900HT Fast Real-Time PCR system (Applied Biosystems) using SYBR green detection chemistry (SYBR Select, Applied Biosystems). The primers were designed using Primer-BLAST (NCBI) and primers either spanned exon-exon junctions or the amplicon spanned exons where the intron region was over one kilobase. Each sample was run in triplicate (see Additional file 2: Table S1 for primer sequences, amplicon lengths, and qRT-PCR reaction parameters). We normalized gene expression relative to a housekeeping gene (*ef1a*) and assessed differential expression using commercial software (REST 2009 [59], Qiagen). Our housekeeping gene has been shown to be stable across sex, tissue types, age, and chemical treatment in zebrafish [60]. Normalizing gene expression to total input RNA produced similar results (Additional file 3: Figure S2). We predicted that qRT-PCR results would follow the RNA-sequencing results and assessed and reported significance using one-tail p-values.

### Behavioral assay

Fish exposed to a stereoisomer of fluoxetine and control fish were tested in the Novel Tank Diving Test (NTDT) using established protocols [11,29] to assess changes in anxiety-related behaviors. Briefly, fish were placed in a 15.2 × 27.9 × 22.5 × 7.1 cm (height × top × bottom × width) trapezoidal tank (Aquatic Ecosystems) filled with 1.4 L of system water. We video-recorded the behavior of the fish for six minutes for later analysis using an animal tracking software package (TopScan Lite, Reston, Virginia, USA). Fish treated with racemic mixture of fluoxetine (n = 12) and control fish (n = 12) were tested for five minutes. All fish were five generations removed from the wild. We report latency to enter the top half of the tank, time spent in top half of the tank and stationary time. Stationary time is measured as the amount of time the fish does not exceed a swimming speed of 0.1 cm/sec [29]. For fish that never entered the top half of the tank we set the latency as the length of the trial. All procedures and protocols in this study were approved by the North Carolina State University Institutional Animal Care and Use Committee.

## Results and discussion

### Fluoxetine reduces anxiety-related behavior but not stereospecifically

**Figure 1:**
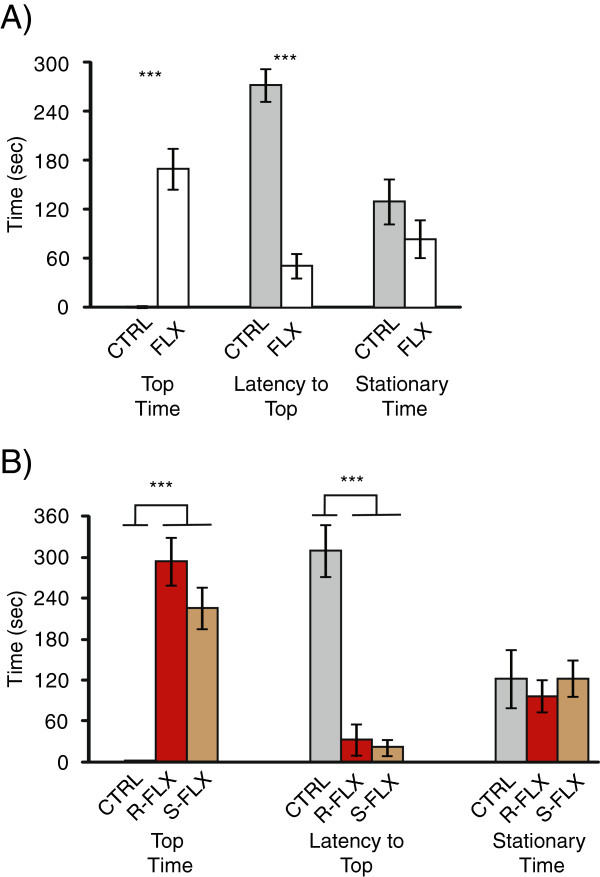
**Behavioral measures after drug treatment. A**) Amount of time spent in the top half of the tank, latency to the top half of the tank and stationary behavior for fish treated with racemic fluoxetine (white) or controls (grey). **B**) Amount of time spent in the top half of the tank, latency to the top half of the tank and stationary behavior for fish treated with R isomer (red), S-isomer (brown) or control (grey). Error bars represent standard error. *, p < 0.05; **, p < 0.01; ***, p < 0.001.

An anxious state is inferred when an animal is placed in an environment where it is conflicted with seeking out potential benefits with that of potential harm (e.g. approach and avoidance) [2]. The approach-avoidance conflict premise is the basis of many common measures of anxiety-related behavioral assays (e.g. open field test, elevated plus maze, light-dark test) [2,61]. In zebrafish one ethologically relevant behavior to potential danger is to swim to the bottom substratum and after the threat has passed, the zebrafish will swim back into the water column. The novel tank diving assay used in this study measures behavioral levels of stress and anxiety-related behaviors in zebrafish by assessing latency to and time spent in the top half of the tank [11,29]. In this study wild-derived zebrafish treated with racemic fluoxetine spent significantly more time in (t = -6.49, p = 2 × 10^-6^) and had a shorter latency to the top half of the tank (t = 8.92, p = 1.38 × 10^-8^) compared to the controls (Figure 1A). This suggests that fluoxetine treatment reduced stress and anxiety-related behaviors in male zebrafish. Our data are consistent with published anxiolytic effects of fluoxetine in domestiecated zebrafish and rodents [11,12]. Fluoxetine has also been documented to alter locomotion [11,12], which could confound interpretations of stress and anxiety levels. In this study we did not observe significant differences in locomotion time (i.e. stationary time (t-stat = 1.29, p = 0.21), Figure 1A) suggesting that in our experiment the anxiolytic effect of fluoxetine is not likely to be a consequence of reduced ability or motivation to swim.

While both isomers reduced stress and anxiety-related behaviors, one was not more potent than the other. There were no significant differences in time spent in the top half of the tank between zebrafish treated with R- or S- fluoxetine (t = 1.47, p = 0.16, Figure 1B), but both treatment groups spent significantly more time in the top than controls (F = 30.13, p = 8.31 × 10^-8^, Figure 1B). Relatedly, fish treated with either isomer had a shorter latency to enter the top half of the tank relative to controls (F = 39.32, p = 5.5 × 10^-9^, Figure 1B) but there were no stereospecific effects (t = 0.39, p = 0.7, Figure 1B). Each fluoxetine isomer had minimal effects on locomotor activity (F = 0.22, p = 0.8, Figure 1B). Our data are consistent with the idea that given both fluoxetine isomers exhibit similar pharmacodynamics in inhibiting the serotonin transporter [6-10], there would be no predicted differences in anxiolytic effects. Fluoxetine, however, increases neurosteroid production (e.g. allopregnanolone) in a stereospecific manner and this has been proposed as an alternative mechanism of action for anxiolytics in mammals [14,62]. Although we did not see any stereospecific anxiolytic effects of fluoxetine at the behavioral level, we have evidence that steroidogenic molecular pathways in the brain are over-represented with racemic treatment (Table 1 and 2, discussion below). Hence it is possible in zebrafish that some anxiolytic mechanisms of fluoxetine include alteration of both serotonin and neurosteroid levels.

**Table 1:** Gene ontology analysis of differentially expressed genes

**Gene ontology**			**FDR corrected p-value**		
**Category**	**Term**	**ID**	**DEG (n=411)**	**Up-regulated (n= 167)**	**Down-regulated (n=244)**
BP	lipid metabolic process	GO:0006629	1.62E-03		6.01E-07
BP	lipid biosynthetic process	GO:0008610	8.22E-03		5.75E-05
BP	fatty acid metabolic process	GO:0006631	8.77E-03		2.21E-04
BP	very long-chain fatty acid metabolic process	GO:0000038	7.78E-03		1.99E-03
BP	fatty acid biosynthetic process	GO:0006633	1.09E-02		4.35E-04
BP	very long-chain fatty acid biosynthetic process	GO:0042761	7.78E-03		1.99E-03
BP	small molecule metabolic process	GO:0044281	1.06E-02		
BP	organic acid metabolic process	GO:0006082	1.56E-04	1.83E-02	
BP	oxoacid metabolic process	GO:0043436	1.56E-04	1.83E-02	
BP	carboxylic acid metabolic process	GO:0019752	5.22E-04	1.43E-02	
BP	single-organism biosynthetic process	GO:0044711	2.24E-03		
BP	small molecule biosynthetic process	GO:0044283	1.93E-03		
BP	organic acid biosynthetic process	GO:0016053	2.86E-04		
BP	carboxylic acid biosynthetic process	GO:0046394	2.86E-04		
BP	L-serine metabolic process	GO:0006563	2.66E-02	2.66E-03	
BP	organonitrogen compound metabolic process	GO:1901564		4.70E-02	
BP	organonitrogen compound biosynthetic process	GO:1901566		1.88E-02	
BP	cellular amino acid metabolic process	GO:0006520		2.27E-03	
BP	cellular amino acid biosynthetic process	GO:0008652		2.54E-02	
BP	alpha-amino acid biosynthetic process	GO:1901607		1.66E-02	
BP	serine family amino acid biosynthetic process	GO:0009070		6.35E-03	
BP	response to virus	GO:0009615		3.05E-03	
BP	monocarboxylic acid metabolic process	GO:0032787			2.07E-03
BP	monocarboxylic acid biosynthetic process	GO:0072330			2.60E-03
BP	steroid metabolic process	GO:0008202			4.99E-02
BP	cellular lipid metabolic process	GO:0044255			4.82E-03
BP	sterol metabolic process	GO:0016125			1.21E-02
MF	phosphorylase activity	GO:0004645	4.24E-02		7.31E-03
MF	glycogen phosphorylase activity	GO:0008184	1.72E-02		2.95E-03
MF	cofactor binding	GO:0048037	3.24E-02		
MF	catalytic activity	GO:0003824	3.19E-02		
MF	oxidoreductase activity	GO:0016491	2.96E-03		1.43E-03
MF	oxidoreductase activity, acting on CH-OH group of donors	GO:0016614			8.66E-03
MF	transferase activity, transferring acyl groups	GO:0016746			9.02E-03
MF	oxidoreductase activity, acting on paired donors, with incorporation or reduction of molecular oxygen, NADH or NADPH as one donor, and incorporation of one atom of oxygen	GO:0016709			2.47E-02

*BP* Biological Process, *MF* Molecular Function, *DEG* all differentially expressed genes. Blank cells indicate p > 0.05.

**Table 2:** KEGG pathway analysis of differentially expressed genes

**KEGG Pathway**	**FDR corrected p-value**
Fatty acid elongation	2.21E-02
Metabolic pathways	5.11E-04
Arachidonic acid metabolism	1.98E-03
Synthesis and degradation of ketone bodies	1.08E-02
Terpenoid backbone biosynthesis	4.36E-03
Glycine, serine and threonine metabolism	2.77E-02
Metabolism of xenobiotics by cytochrome P450	6.78E-03
Steroid biosynthesis	1.96E-09
Steroid hormone biosynthesis	1.27E-03

### Changes in gene expression consistent with alterations in stress and anxiety levels

**Figure 2:**
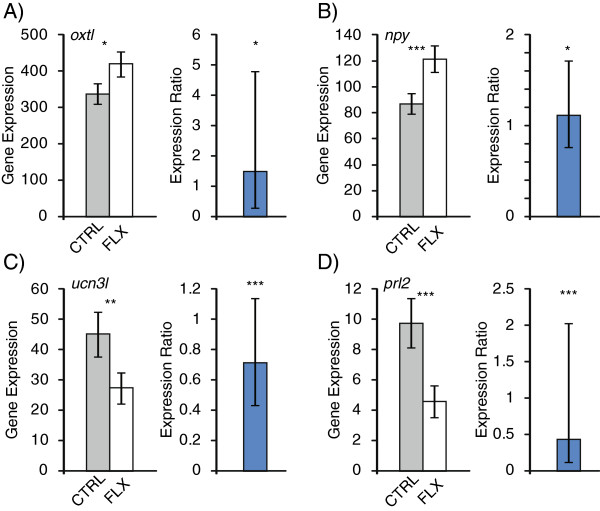
**RNA-sequencing and qRT-PCR expression of select genes with racemic fluoxetine treatment.** Gene expression of (**A**) isotocin, (**B**) neuropeptide Y, (**C**) urocortin 3, and (**D**) prolactin. In each panel left and right graphs represent quantification from RNA-sequencing (fragments per kilobase per million reads, FPKM) and qRT-PCR (expression ratio: gene expression in fluoxetine treatment / control), respectively. qRT-PCR gene expression was normalized to a housekeeping gene. Grey and white bars represent control and fluoxetine treatment, respectively. Error bars represent 95% confidence intervals. *, p < 0.05; **, p < 0.01; ***, p < 0.001.

Neuropeptides have modulatory roles in a variety of behaviors including stress and anxiety. RNA-sequencing analysis showed that several neuropeptides associated with stress and anxiety in other studies were differentially regulated with racemic fluoxetine treatment and validated with qRT-PCR (Figure 2). Isotocin (*oxtl*, RNA-sequencing: test statistic = -3.74, p = 0.014; qRT-PCR: p = 0.034) and neuropeptide Y (*npy*, test statistic = -5.37, p = 1.8 × 10^-5^; qRT-PCR: p = 0.045) showed significantly higher expression in fluoxetine treated fish relative to controls (Figure 2A, B). While isotocin is homologous to the mammalian oxytocin, relatively little is known about isotocin’s behavioral functions in fishes [63]. In wild-type zebrafish, peripheral isotocin injection decreased the level of fear response to a predator in a dose-dependent manner [64], which suggests an anxiolytic effect. In rodents, pharmacological manipulations, gene expression, and transgenic animal studies have shown increases in oxytocin and NPY levels are associated with reduced anxiety [65-72]. Oxytocin is thought to affect many social behaviors in humans [73] including having anxiolytic effects [74,75]. Interestingly, variants of NPY in humans are associated with anxiety susceptibility [76] and zebrafish may be an ideal system to explore this further.

Anxiety-related behavioral displays are often associated with a physiological stress response. The stress response in teleosts has been well-described [77,78] and the molecular mechanisms underlying the cortisol stress response in zebrafish are similar to mammals [33,79]. While we did not measure corticosteroid levels, the first study administering fluoxetine to domesticated zebrafish in the same paradigm used in this study found reduced whole-body cortisol levels compared to controls [11]. At the molecular level, urocortin 3 (*ucn3l*, part of the corticotrophin-releasing hormone family) and prolactin (*prl2*) were down-regulated with fluoxetine treatment in both RNA-sequencing (*ucn3l*: test statistic = 3.99, p = 0.006; *prl2*: test statistic = 5.35, p = 1.98 × 10^-5^; Figure 2C, D) and qRT-PCR analyses (*ucn3l* and *prl2*, p < 0.001, Figure 2C, D). Urocortin 1-3 interact with the corticotrophin system in the brain by binding to the same receptors and can alter stress and anxiety [80-83]. Specifically, central administration of urocortins has been shown to be anxiogenic in rats [84-88]. The exact role of urocortin 3 in mediating stress and anxiety is not well understood as pharmacological and knock-out studies in rodents produce inconsistent results [89-91]. Prolactin in the brain is often associated with maternal behavior, but it is also associated with stress coping [92-94]. Other teleosts displayed elevated plasma prolactin levels in response to stress [95,96], which is consistent with our results where treatment with an anxiolytic reduced *prl2* expression. It should be noted that for the gene expression parts of this study, we did not induce stress prior to brain extraction and this may explain why more genes associated with a stress response were not differentially regulated (Additional file 4: Table S2). Hence the reduction of *ucn3l* and *prl2* expression compared to the controls is consistent with the fluoxetine-treated fish having lower basal stress levels.

**Figure 3:**
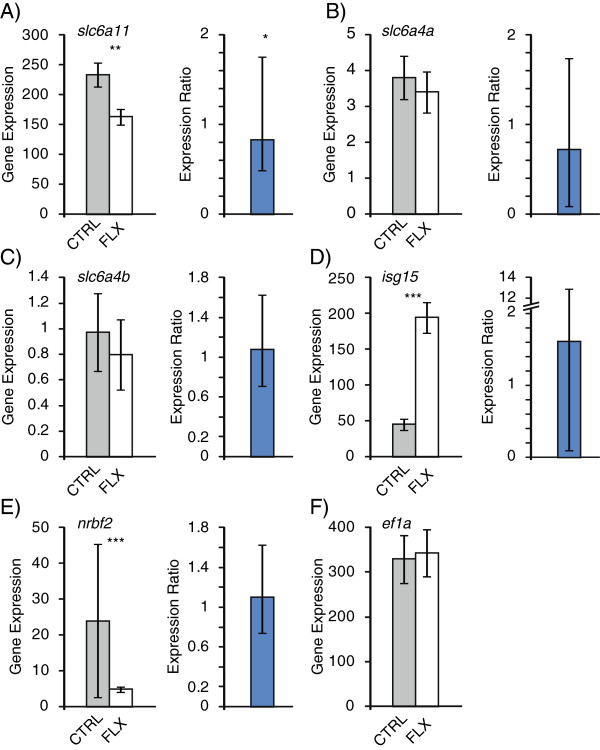
**RNA-sequencing and qRT-PCR expression of select genes with racemic fluoxetine treatment.** Gene expression of (**A**) GABA transporter, (**B**) serotonin transporter A, (**C**) serotonin transporter B, (**D**) ISG15 ubiquitin-like modifier, (**E**) nuclear receptor binding factor 2, and (**F**) elongation factor 1-alpha. In each panel left and right graphs represent quantification from RNA-sequencing (fragments per kilobase per million reads, FPKM) and qRT-PCR (expression ratio: gene expression in fluoxetine treatment / control), respectively. qRT-PCR gene expression was normalized to a housekeeping gene. Grey and white bars represent control and fluoxetine treatment, respectively. Error bars represent 95% confidence intervals. *, p < 0.05; **, p < 0.01; ***, p < 0.001.

Neurotransmitter transporters have an important role in maintaining neural activity. The gene coding for the GABA transporter, *slc6a11*, was significantly down-regulated in fluoxetine treated fish with RNA-sequencing (test statistic = 6.06, p = 4.49 × 10^-7^, Figure 3A). Alteration of GABA mediated signaling is one proposed causal mechanism of anxiety and GABA transporter knockout mice exhibit reduced anxiety- and depression-related behaviors [97]. Hence, lower expression of GABA transporter is consistent with the finding that fluoxetine treated fish had reduced stress and anxiety-related behaviors. qRT-PCR validation showed mixed results where normalizing to a housekeeping gene showed significant differences between groups (p = 0.035, Figure 3A) but normalizing to total RNA did not (t = 1.34, p = 0.096, Additional file 3: Figure S2). The serotonin transporters (*slc6a4a* and *slc6a4b*) showed no differences in expression between groups by RNA-sequencing (*slc6a4a*: test statistic = 0.957, p = 0.855; *slc6a4b*: test statistic = 0.86, p = 0.876; Figure 3B, C) or qRT-PCR (*slc6a4a*: p = 0.058; *slc6a4b*: p = 0.242; Figure 3B, C). This is consistent with other studies in teleosts and rodents where chronic fluoxetine treatment altered expression of genes other than the serotonin transporter [42,98-101]. SSRIs bind to serotonin transporters to inhibit their function, however, SSRIs may not regulate the expression of the transporter. Although several microarray studies focusing on a specific brain region did not show significant differences in expression of the serotonin transporter gene with fluoxetine treatment [98-101], we cannot rule out brain region specific regulation in zebrafish. Two genes (*isg15*, *nrbf2*) showed greater than two fold differences in expression between groups in RNA-sequencing (Figure 3D, E, Additional file 4: Table S2) but were not validated by qPCR (*isg15*: p = 0.1; *nrbf2*: p = 0.072). The housekeeping gene, elongation factor 1-alpha (*ef1a*) showed no significant difference between groups in either RNA-sequencing (test statistic = 0.258, p = 0.976, Figure 3F) or qRT-PCR (Additional file 3: Figure S2). Overall, 80% of the genes we examined by qRT-PCR showed consistent expression patterns as RNA-sequencing, which is a rate consistent with other transcriptome studies [102,103]. There was a trend for a positive correlation between RNA-sequencing and qRT-PCR expression levels across all genes (n = 10, r = 0.49, p = 0.072). However, when excluding genes (*isg15*, *nrbf2*) that did not show consistent expression patterns between RNA-sequencing and qRT-PCR, there was a significant positive correlation (n = 8, r = 0.88, p = 0.002).

### Identification of global transcriptome changes and related pathways

**Figure 4:**
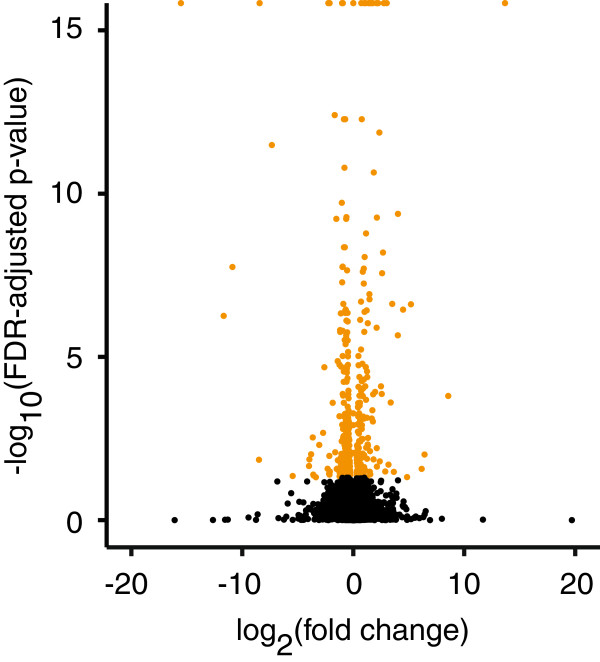
**Volcano plot of all protein-coding genes analyzed by RNA-sequencing.** Genes differentially expressed with p < 0.05 after correcting for false discovery rate are in orange. Genes with a p > 0.05 after correcting for false discovery rate are in black. Any point above 15 on the y-axis has a p < 5 x 10^-15^.

Of the 26,585 protein-coding genes analyzed, 411 were differentially regulated with racemic fluoxetine treatment (Figure 4, Additional file 4: Table S2). In racemic fluoxetine treated fish, 167 and 244 genes were up- and down-regulated, respectively. Gene ontology (GO) analysis of all differentially expressed genes reveal that terms related to lipid (p = 0.001) and small molecule metabolism (p = 0.01) were significantly over-enriched (Table 1). Interestingly when we accounted for direction of expression in the GO analysis, amino acid and lipid metabolic processes were up-regulated and down-regulated, respectively, with fluoxetine treatment (Table 1). These data suggest that male zebrafish treated with fluoxetine (i.e., reduced anxiety and stress levels) may be shifting macromolecule resources from energy (e.g. lipid) to enzymatic (e.g. amino acid) processes in the brain. It is well known that individuals will change foraging habits in the presence of predators [104,105] and it is plausible that less anxious zebrafish seek out foods higher in protein content. While we fed the fluoxetine-treated and control groups *ad libitum* future studies should assess for differences in food consumption or diet preference. An equally likely hypothesis is that individuals with higher stress and anxiety levels have disrupted neurosteroid levels - possibly due to altered precursor activity. Of note, fish used in this study were selectively bred to exhibit high levels of stress and anxiety behaviors and hence control fish are presumed to have high levels of stress and anxiety [29]. Highly stressed and anxious (e.g., control) fish have up-regulated lipid and steroid metabolism (Table 1) that may lead to altered neurosteroid production (via precursor availability). We acknowledge that transcriptome wide patterns are challenging to interpret and in this study we can only speculate on the precise roles of lipid and amino acid metabolism in stress and anxiety. It is also possible that by utilizing the whole brain, we may not be able to detect some pathways that are differentially expressed in specific regions. It should be noted, however, that whole-brain transcriptome studies still provide important insights into the molecular mechanisms of sex differences and a variety of social behaviors [56,106-114]. Future studies should use transgenic zebrafish or pharmacologically alter expression levels to better characterize the role of a gene. Complementary studies could also assess localized differential expression patterns of “candidate” genes across the brain via neurohistochemical techniques [115-117] to assist in identifying candidate brain regions for transcriptomic analyses. Alternatively, using zebrafish with naturally high and low stress and anxiety-related behavioral levels (e.g., HSB, LSB [29]) will provide an opportunity to test for similar sorts of transcriptome changes in a non-pharmacological context and is currently underway in our laboratory.

Using an alternative database to analyze global transcriptome changes that has associations with pathways, Kyoto Encyclopedia of Genes and Genomes (KEGG [118,119]), resulted in largely similar over-represented pathways as GO analysis (Table 2). Amino acid pathways (glycine, serine, and threonine, p = 5.11 × 10^-4^) and lipid related pathways (fatty acid elongation, p = 0.022) were over-represented (Table 2). Notably, steroid (p = 1.96 × 10^-9^) and steroid hormone (p = 0.001) biosynthesis pathways were also over-represented (Table 2). While we did not measure steroid levels in this study, in male goldfish treated with fluoxetine, there was an increase in estradiol, decrease in testosterone, and no changes in luteinizing or growth hormone serum levels but how these changes relate to anxiety-related behaviors was not discussed [120]. Neurosteroids (e.g. allopregnanolone) are implicated as potential mediators of anxiety in mammals [13-15,18,121]. Fluoxetine can increase neurosteroid content and S-fluoxetine increases allopregnanolone levels more effectively than the R isomer [13,14,21]. Allopregnanolone reduces anxiety in rodents and has a similar efficacy as fluoxetine [122-125]. As we see reduction in anxiety-related behaviors (Figure 1) and over-representation of steroid hormone pathways (Table 2) in the brain with fluoxetine treatment, it is possible that the anxiolytic effects seen in this study are due to changes in neurosteroid content.

Few studies have examined transcriptome level changes in gene expression with fluoxetine treatment in teleosts. One study has examined the effects of fluoxetine treatment on differential gene expression in whole body larval zebrafish through microarrays [42]. Of genes we found differentially expressed in adult male whole-brain zebrafish, only three of these genes (*fkbp5*, *itm2cb*, and an unannotated gene) were also differentially expressed in larval zebrafish [42]. Fluoxetine is known to have age-dependent effects in mammals and it is possible that we are observing molecular evidence of this in zebrafish [126-128]. It should be noted though, that changes in membrane protein activity and/or composition (e.g. *fkbp5*, *itm2cb*) may be a common mechanism of fluoxetine action across the lifecycle. Interestingly, in a microarray study identifying hypothalamic differential gene expression in female goldfish treated with fluoxetine, we observed only three genes (*trib3*, *oxtl*, *mbpb*) that were similarly differentially regulated in our study. It is possible that this minimal amount of overlap between lists of genes differentially regulated is due to sex or regional specificity. Hypothalamic isotocin levels decreased with fluoxetine treatment in female goldfish [98] but showed no expression differences in male telencephalon [120]. It should be noted that neither of the studies utilizing fluoxetine-treated goldfish [98,120] or larval zebrafish [42] measured anxiety-related behavior. Surprisingly, the lack of a large overlap of differentially expressed genes with fluoxetine treatment was also found between mice and rats with genomic analyses [99-101]. While there may be species- and brain region-specific effects of fluoxetine, uncovering potential universal mechanisms of action of fluoxetine will need to utilize a more comprehensive comparative approach.

## Conclusions

In this study we present data from both behavioral and molecular levels showing that fluoxetine alters stress and anxiety-related behaviors in wild-derived male zebrafish. Racemic and stereoisomers of fluoxetine reduce anxiety-related behaviors as indicated by drug treated fish spending more time in the top half of the tank. There were no stereoisomer effects on behavior but these effects were not tested at the molecular levels where it is possible that GABAergic and serotonergic activity were altered. In the small sampling of “candidate” neuropeptides (*npy*, *oxtl*, *prl2*, *ucn3l*) linked to stress and anxiety in the literature, our gene expression results were consistent with fluoxetine-induced changes at the molecular level in other model systems. Larger scale analyses reveal that lipid and amino acid metabolic processes and steroid biosynthesis are disproportionately influenced. Changes in lipid, amino acid and neurosteroid utilization in the brain may be characteristic of fluoxetine treatment and, by extension, stress and anxiety.

## Competing interests

The authors declare that they have no competing interests.

## Authors’ contributions

RYW carried out the transcriptome and qRT-PCR studies, participated in the behavioral experiments, analyzed and performed statistical analyses on all data, participated in the design of the experiment, and drafted the manuscript. SEO carried out and quantified the behavioral experiments for the stereoisomers of fluoxetine. JG conceived the study, participated in the design of the experiment, interpretation of the data, and helped to draft the manuscript. All authors read and approved the final manuscript.

## Supplementary Material

Additional file 1: Figure S1 Fluoxetine dose-response curve. Time spent on the top half of the tank (y-axis) for fish treated with 0, 11, 33, and 100 μg/L racemic fluoxetine (x-axis). N = 6 at each concentration. Click here for file

Additional file 2: Table S1 qRT-PCR primer characteristics. Click here for file

Additional file 3: Figure S2 qRT-PCR validation of expression of select genes with racemic fluoxetine treatment. Genes examined are (A) isotocin, (B) neuropeptide Y, (C) urocortin 3, (D), prolactin, (E) GABA transporter, (F) serotonin transporter A, (G) serotonin transporter B, (H) ISG15 ubiquitin-like modifier, (I) nuclear receptor binding factor 2, and (J) elongation factor 1-alpha. Gene expression values are normalized to total RNA input. Error bars represent standard error. *, p < 0.05; **; ***, p < 0.001; +, data is consistent with RNA-sequencing results. Click here for file

Additional file 4: Table S2 Significant differentially expressed genes with fluoxetine treatment after correcting for false discovery rate. Click here for file

## References

[B1] American Psychiatric Association Diagnostic criteria from DSM-IV-TR 2000 Washington, D.C: American Psychiatric Association

[B2] Cryan JF Sweeney FF The age of anxiety: role of animal models of anxiolytic action in drug discovery Br J Pharmacol 2011 164 1129 1161 10.1111/j.1476-5381.2011.01362.x 21545412 PMC3229755

[B3] Durant C Christmas D Nutt D The pharmacology of anxiety Curr Top Behav Neurosci 2010 2 303 330 21309115 10.1007/7854_2009_8

[B4] Jacobson LH Cryan JF Genetic approaches to modeling anxiety in animals Curr Top Behav Neurosci 2010 2 161 201 21309110 10.1007/7854_2009_31

[B5] Westenberg HG Recent advances in understanding and treating social anxiety disorder CNS Spectr 2009 14 24 33 19238127 10.1017/s1092852900027267

[B6] Pinna G Costa E Guidotti A Fluoxetine and norfluoxetine stereospecifically facilitate pentobarbital sedation by increasing neurosteroids Proc Natl Acad Sci USA 2004 101 6222 6225 10.1073/pnas.0401479101 15069199 PMC395950

[B7] Wong DT Bymaster FP Engleman EA Prozac (fluoxetine, lilly 110140), the first selective serotonin uptake inhibitor and an antidepressant drug: Twenty years since its first publication Life Sci 1995 57 411 441 10.1016/0024-3205(95)00209-O 7623609

[B8] Wong DT Bymaster FP Reid LR Fuller RW Perry KW Inhibition of serotonin uptake by optical isomers of fluoxetine Drug Dev Res 1985 6 397 403 10.1002/ddr.430060412

[B9] Koch S Perry KW Nelson DL Conway RG Threlkeld PG Bymaster FP R-fluoxetine increases extracellular DA, NE, as well as 5-HT in rat prefrontal cortex and hypothalamus: an in vivo microdialysis and receptor binding study Neuropsychopharmacology 2002 27 949 959 10.1016/S0893-133X(02)00377-9 12464452

[B10] Robertson DW Krushinski JH Fuller RW Leander JD Absolute configurations and pharmacological activities of the optical isomers of fluoxetine, a selective serotonin-uptake inhibitor J Med Chem 1988 31 1412 1417 10.1021/jm00402a027 3260286

[B11] Egan RJ Bergner CL Hart PC Cachat JM Canavello PR Elegante MF Elkhayat SI Bartels BK Tien AK Tien DH Understanding behavioral and physiological phenotypes of stress and anxiety in zebrafish Behav Brain Res 2009 205 38 44 10.1016/j.bbr.2009.06.022 19540270 PMC2922906

[B12] Dulawa SC Holick KA Gundersen B Hen R Effects of Chronic Fluoxetine in Animal Models of Anxiety and Depression Neuropsychopharmacology 2004 29 1321 1330 10.1038/sj.npp.1300433 15085085

[B13] Pinna G Costa E Guidotti A SSRIs act as selective brain steroidogenic stimulants (SBSSs) at low doses that are inactive on 5-HT reuptake Curr Opin Pharmacol 2009 9 24 30 10.1016/j.coph.2008.12.006 19157982 PMC2670606

[B14] Pinna G Costa E Guidotti A Fluoxetine and norfluoxetine stereospecifically and selectively increase brain neurosteroid content at doses that are inactive on 5-HT reuptake Psychopharmacology (Berl) 2006 186 362 372 10.1007/s00213-005-0213-2 16432684

[B15] Longone P Di Michele F D'Agati E Romeo E Pasini A Rupprecht R Neurosteroids as neuromodulators in the treatment of anxiety disorders Front Endocrinol (Lausanne) 2011 2 55 22654814 10.3389/fendo.2011.00055 PMC3356011

[B16] Barbaccia ML Neurosteroidogenesis: relevance to neurosteroid actions in brain and modulation by psychotropic drugs Crit Rev Neurobiol 2004 16 67 74 10.1615/CritRevNeurobiol.v16.i12.70 15581401

[B17] Reddy DS O'Malley BW Rogawski MA Anxiolytic activity of progesterone in progesterone receptor knockout mice Neuropharmacology 2005 48 14 24 10.1016/j.neuropharm.2004.09.002 15617723

[B18] Pinna G Rasmusson AM Up-regulation of neurosteroid biosynthesis as a pharmacological strategy to improve behavioural deficits in a putative mouse model of post-traumatic stress disorder J Neuroendocrinol 2012 24 102 116 10.1111/j.1365-2826.2011.02234.x 21981145 PMC3245370

[B19] Schule C Eser D Baghai TC Nothdurfter C Kessler JS Rupprecht R Neuroactive steroids in affective disorders: target for novel antidepressant or anxiolytic drugs? Neuroscience 2011 191 55 77 21439354 10.1016/j.neuroscience.2011.03.025

[B20] Guidotti A Dong E Matsumoto K Pinna G Rasmusson AM Costa E The socially-isolated mouse: a model to study the putative role of allopregnanolone and 5Î±-dihydroprogesterone in psychiatric disorders Brain Res Rev 2001 37 110 115 10.1016/S0165-0173(01)00129-1 11744079

[B21] Pinna G Dong E Matsumoto K Costa E Guidotti A In socially isolated mice, the reversal of brain allopregnanolone down-regulation mediates the anti-aggressive action of fluoxetine Proc Natl Acad Sci USA 2003 100 2035 2040 10.1073/pnas.0337642100 12571361 PMC149954

[B22] Jain NS Hirani K Chopde CT Reversal of caffeine-induced anxiety by neurosteroid 3-alpha-hydroxy-5-alpha-pregnane-20-one in rats Neuropharmacology 2005 48 627 638 10.1016/j.neuropharm.2004.11.016 15814098

[B23] Bitran D Foley M Audette D Leslie N Frye CA Activation of peripheral mitochondrial benzodiazepine receptors in the hippocampus stimulates allopregnanolone synthesis and produces anxiolytic-like effects in the rat Psychopharmacology (Berl) 2000 151 64 71 10.1007/s002130000471 10958118

[B24] Landgraf R Neuropeptides in anxiety modulation Handb Exp Pharmacol 2005 169 335 369 10.1007/3-540-28082-0_12 16594264

[B25] Semsar K Perreault HA Godwin J Fluoxetine-treated male wrasses exhibit low AVT expression Brain Res 2004 1029 141 147 10.1016/j.brainres.2004.09.030 15542067

[B26] Cryan JF Holmes A The ascent of mouse: advances in modelling human depression and anxiety Nat Rev Drug Discov 2005 4 775 790 10.1038/nrd1825 16138108

[B27] Overli O Pottinger TG Carrick TR Overli E Winberg S Differences in behaviour between rainbow trout selected for high- and low-stress responsiveness J Exp Biol 2002 205 391 395 11854375 10.1242/jeb.205.3.391

[B28] Landgraf R Wigger A High vs low anxiety-related behavior rats: an animal model of extremes in trait anxiety Behav Genet 2002 32 301 314 10.1023/A:1020258104318 12405513

[B29] Wong RY Perrin F Oxendine SE Kezios ZD Sawyer S Zhou L Dereje S Godwin J Comparing behavioral responses across multiple assays of stress and anxiety in zebrafish (Danio rerio) Behaviour 2012 149 1205 1240 10.1163/1568539X-00003018

[B30] Maximino C de Brito TM da Silva Batista AW Herculano AM Morato S Gouveia A Jr Measuring anxiety in zebrafish: a critical review Behav Brain Res 2010 214 157 171 10.1016/j.bbr.2010.05.031 20510300

[B31] Stewart A Gaikwad S Kyzar E Green J Roth A Kalueff AV Modeling anxiety using adult zebrafish: a conceptual review Neuropharmacology 2012 62 135 143 10.1016/j.neuropharm.2011.07.037 21843537 PMC3195883

[B32] Clark KJ Boczek NJ Ekker SC Stressing zebrafish for behavioral genetics Rev Neurosci 2011 22 49 62 21615261 10.1515/RNS.2011.007 PMC3470424

[B33] Steenbergen PJ Richardson MK Champagne DL The use of the zebrafish model in stress research Prog Neuropsychopharmacol Biol Psychiatry 2011 35 1432 1451 10.1016/j.pnpbp.2010.10.010 20971150

[B34] Champagne DL Hoefnagels CC de Kloet RE Richardson MK Translating rodent behavioral repertoire to zebrafish (Danio rerio): relevance for stress research Behav Brain Res 2010 214 332 342 10.1016/j.bbr.2010.06.001 20540966

[B35] Howe K Clark MD Torroja CF Torrance J Berthelot C Muffato M Collins JE Humphray S McLaren K Matthews L The zebrafish reference genome sequence and its relationship to the human genome Nature 2013 496 498 503 10.1038/nature12111 23594743 PMC3703927

[B36] Griebel G Belzung C Perrault G Sanger DJ Differences in anxiety-related behaviours and in sensitivity to diazepam in inbred and outbred strains of mice Psychopharmacology (Berl) 2000 148 164 170 10.1007/s002130050038 10663431

[B37] Miller BH Schultz LE Gulati A Su AI Pletcher MT Phenotypic Characterization of a Genetically Diverse Panel of Mice for Behavioral Despair and Anxiety PLoS One 2010 5 e14458 10.1371/journal.pone.0014458 21206921 PMC3012073

[B38] Panula P Chen YC Priyadarshini M Kudo H Semenova S Sundvik M Sallinen V The comparative neuroanatomy and neurochemistry of zebrafish CNS systems of relevance to human neuropsychiatric diseases Neurobiol Dis 2010 40 46 57 10.1016/j.nbd.2010.05.010 20472064

[B39] Lillesaar C The serotonergic system in fish J Chem Neuroanat 2011 41 294 308 10.1016/j.jchemneu.2011.05.009 21635948

[B40] Wang Y Takai R Yoshioka H Shirabe K Characterization and expression of serotonin transporter genes in zebrafish Tohoku J Exp Med 2006 208 267 274 10.1620/tjem.208.267 16498236

[B41] Gould GG Brooks BW Frazer A: [(3)H] citalopram binding to serotonin transporter sites in minnow brains Basic Clin Pharmacol Toxicol 2007 101 203 210 10.1111/j.1742-7843.2007.00100.x 17697042

[B42] Park JW Heah TP Gouffon JS Henry TB Sayler GS Global gene expression in larval zebrafish (Danio rerio) exposed to selective serotonin reuptake inhibitors (fluoxetine and sertraline) reveals unique expression profiles and potential biomarkers of exposure Environ Pollut 2012 167 163 170 22575097 10.1016/j.envpol.2012.03.039

[B43] Zon LI Peterson RT In vivo drug discovery in the zebrafish Nat Rev Drug Discov 2005 4 35 44 10.1038/nrd1606 15688071

[B44] Rihel J Schier AF Behavioral screening for neuroactive drugs in zebrafish Dev Neurobiol 2012 72 373 385 10.1002/dneu.20910 21567979

[B45] Grunwald DJ Eisen JS Headwaters of the zebrafish – emergence of a new model vertebrate Nat Rev Genet 2002 3 717 724 12209146 10.1038/nrg892

[B46] Brooks BW Chambliss CK Stanley JK Ramirez A Banks KE Johnson RD Lewis RJ Determination of select antidepressants in fish from an effluent-dominated stream Environ Toxicol Chem 2005 24 464 469 10.1897/04-081R.1 15720009

[B47] Gaworecki KM Klaine SJ Behavioral and biochemical responses of hybrid striped bass during and after fluoxetine exposure Aquat Toxicol 2008 88 207 213 10.1016/j.aquatox.2008.04.011 18547660

[B48] Paterson G Metcalfe CD Uptake and depuration of the anti-depressant fluoxetine by the Japanese medaka (Oryzias latipes) Chemosphere 2008 74 125 130 10.1016/j.chemosphere.2008.08.022 18845313

[B49] Auer PL Doerge RW Statistical design and analysis of RNA sequencing data Genetics 2010 185 405 416 10.1534/genetics.110.114983 20439781 PMC2881125

[B50] Wu TD Nacu S Fast and SNP-tolerant detection of complex variants and splicing in short reads Bioinformatics 2010 26 873 881 10.1093/bioinformatics/btq057 20147302 PMC2844994

[B51] Trapnell C Roberts A Goff L Pertea G Kim D Kelley DR Pimentel H Salzberg SL Rinn JL Pachter L Differential gene and transcript expression analysis of RNA-seq experiments with TopHat and Cufflinks Nat Protocols 2012 7 562 578 10.1038/nprot.2012.016 PMC3334321 22383036

[B52] Trapnell C Williams BA Pertea G Mortazavi A Kwan G van Baren MJ Salzberg SL Wold BJ Pachter L Transcript assembly and quantification by RNA-Seq reveals unannotated transcripts and isoform switching during cell differentiation Nat Biotech 2010 28 511 515 10.1038/nbt.1621 PMC3146043 20436464

[B53] Trapnell C Hendrickson DG Sauvageau M Goff L Rinn JL Pachter L Differential analysis of gene regulation at transcript resolution with RNA-seq Nat Biotech 2013 31 46 53 10.1038/nbt.2450 PMC3869392 23222703

[B54] Reimand J Arak T Vilo J g Profiler–a web server for functional interpretation of gene lists (2011 update) Nucleic Acids Res 2011 39 W307 W315 10.1093/nar/gkr378 21646343 PMC3125778

[B55] Reimand J Kull M Peterson H Hansen J Vilo J g Profiler–a web-based toolset for functional profiling of gene lists from large-scale experiments Nucleic Acids Res 2007 35 W193 W200 10.1093/nar/gkm226 17478515 PMC1933153

[B56] Cummings ME Larkins-Ford J Reilly CR Wong RY Ramsey M Hofmann HA Sexual and social stimuli elicit rapid and contrasting genomic responses Proc Biol Sci 2008 275 393 402 10.1098/rspb.2007.1454 18055387 PMC2212751

[B57] Lynch KS Ramsey ME Cummings ME The mate choice brain: comparing gene profiles between female choice and male coercive poeciliids Genes Brain Behav 2012 11 222 229 10.1111/j.1601-183X.2011.00742.x 22008245

[B58] Ramsey ME Maginnis TL Wong RY Brock C Cummings ME Identifying context-specific gene profiles of social, reproductive, and mate preference behavior in a fish species with female mate choice Front Neurosci 2012 6 62 22557945 10.3389/fnins.2012.00062 PMC3340895

[B59] Pfaffl MW Horgan GW Dempfle L Relative expression software tool (REST) for group-wise comparison and statistical analysis of relative expression results in real-time PCR Nucleic Acids Res 2002 30 e36 10.1093/nar/30.9.e36 11972351 PMC113859

[B60] McCurley AT Callard GV Characterization of housekeeping genes in zebrafish: male-female differences and effects of tissue type, developmental stage and chemical treatment BMC Mol Biol 2008 9 102 10.1186/1471-2199-9-102 19014500 PMC2588455

[B61] Sousa N Almeida OF Wotjak CT A hitchhiker’s guide to behavioral analysis in laboratory rodents Genes Brain Behav 2006 5 Suppl 2 5 24 16681797 10.1111/j.1601-183X.2006.00228.x

[B62] Uzunova V Sheline Y Davis JM Rasmusson A Uzunov DP Costa E Guidotti A Increase in the cerebrospinal fluid content of neurosteroids in patients with unipolar major depression who are receiving fluoxetine or fluvoxamine Proc Natl Acad Sci USA 1998 95 3239 3244 10.1073/pnas.95.6.3239 9501247 PMC19726

[B63] Godwin J Thompson R Nonapeptides and social behavior in fishes Horm Behav 2012 61 230 238 10.1016/j.yhbeh.2011.12.016 22285647

[B64] Braida D Donzelli A Martucci R Capurro V Busnelli M Chini B Sala M Neurohypophyseal hormones manipulation modulate social and anxiety-related behavior in zebrafish Psychopharmacology (Berl) 2012 220 319 330 10.1007/s00213-011-2482-2 21956239

[B65] Thorsell A Central neuropeptide Y in anxiety- and stress-related behavior and in ethanol intake Ann N Y Acad Sci 2008 1148 136 140 10.1196/annals.1410.083 19120101

[B66] Neumann ID Landgraf R Balance of brain oxytocin and vasopressin: implications for anxiety, depression, and social behaviors Trends Neurosci 2012 35 649 659 10.1016/j.tins.2012.08.004 22974560

[B67] Karlsson RM Holmes A Heilig M Crawley JN Anxiolytic-like actions of centrally-administered neuropeptide Y, but not galanin, in C57BL/6J mice Pharmacol Biochem Behav 2005 80 427 436 10.1016/j.pbb.2004.12.009 15740785

[B68] Slattery DA Neumann ID Chronic icv oxytocin attenuates the pathological high anxiety state of selectively bred Wistar rats Neuropharmacology 2010 58 56 61 10.1016/j.neuropharm.2009.06.038 19589349

[B69] Kask A Harro J von Horsten S Redrobe JP Dumont Y Quirion R The neurocircuitry and receptor subtypes mediating anxiolytic-like effects of neuropeptide Y Neurosci Biobehav Rev 2002 26 259 283 10.1016/S0149-7634(01)00066-5 12034130

[B70] Missig G Ayers LW Schulkin J Rosen JB Oxytocin reduces background anxiety in a fear-potentiated startle paradigm Neuropsychopharmacology 2010 35 2607 2616 10.1038/npp.2010.155 20844476 PMC3055566

[B71] Trent NL Menard JL Infusions of neuropeptide Y into the lateral septum reduce anxiety-related behaviors in the rat Pharmacol Biochem Behav 2011 99 580 590 10.1016/j.pbb.2011.06.009 21693128

[B72] Onaka T Takayanagi Y Yoshida M Roles of oxytocin neurones in the control of stress, energy metabolism, and social behaviour J Neuroendocrinol 2012 24 587 598 10.1111/j.1365-2826.2012.02300.x 22353547

[B73] Meyer-Lindenberg A Domes G Kirsch P Heinrichs M Oxytocin and vasopressin in the human brain: social neuropeptides for translational medicine Nat Rev Neurosci 2011 12 524 538 10.1038/nrn3044 21852800

[B74] Labuschagne I Phan KL Wood A Angstadt M Chua P Heinrichs M Stout JC Nathan PJ Oxytocin attenuates amygdala reactivity to fear in generalized social anxiety disorder Neuropsychopharmacology 2010 35 2403 2413 10.1038/npp.2010.123 20720535 PMC3055328

[B75] Petrovic P Kalisch R Singer T Dolan RJ Oxytocin attenuates affective evaluations of conditioned faces and amygdala activity J Neurosci 2008 28 6607 6615 10.1523/JNEUROSCI.4572-07.2008 18579733 PMC2647078

[B76] Donner J Sipila T Ripatti S Kananen L Chen X Kendler KS Lonnqvist J Pirkola S Hettema JM Hovatta I Support for involvement of glutamate decarboxylase 1 and neuropeptide Y in anxiety susceptibility Am J Med Genet B Neuropsychiatr Genet 2012 159B 316 327 10.1002/ajmg.b.32029 22328461

[B77] Wendelaar Bonga SE The stress response in fish Physiol Rev 1997 77 591 625 9234959 10.1152/physrev.1997.77.3.591

[B78] Barton BA Stress in fishes: a diversity of responses with particular reference to changes in circulating corticosteroids Integr Comp Biol 2002 42 517 525 10.1093/icb/42.3.517 21708747

[B79] Alsop D Vijayan MM Molecular programming of the corticosteroid stress axis during zebrafish development Comp Biochem Physiol A Mol Integr Physiol 2009 153 49 54 10.1016/j.cbpa.2008.12.008 19146973

[B80] Fekete EM Zorrilla EP Physiology, pharmacology, and therapeutic relevance of urocortins in mammals: ancient CRF paralogs Front Neuroendocrinol 2007 28 1 27 10.1016/j.yfrne.2006.09.002 17083971 PMC2730896

[B81] Pan W Kastin AJ Urocortin and the brain Prog Neurobiol 2008 84 148 156 10.1016/j.pneurobio.2007.10.008 18078706 PMC2267723

[B82] Gysling K Forray MI Haeger P Daza C Rojas R Corticotropin-releasing hormone and urocortin: redundant or distinctive functions? Brain Res Brain Res Rev 2004 47 116 125 10.1016/j.brainresrev.2004.06.001 15572167

[B83] Neufeld-Cohen A Tsoory MM Evans AK Getselter D Gil S Lowry CA Vale WW Chen A A triple urocortin knockout mouse model reveals an essential role for urocortins in stress recovery Proc Natl Acad Sci USA 2010 107 19020 19025 10.1073/pnas.1013761107 20937857 PMC2973872

[B84] Spina MG Merlo-Pich E Akwa Y Balducci C Basso AM Zorrilla EP Britton KT Rivier J Vale WW Koob GF Time-dependent induction of anxiogenic-like effects after central infusion of urocortin or corticotropin-releasing factor in the rat Psychopharmacology (Berl) 2002 160 113 121 10.1007/s00213-001-0940-y 11875628

[B85] Gehlert DR Shekhar A Morin SM Hipskind PA Zink C Gackenheimer SL Shaw J Fitz SD Sajdyk TJ Stress and central Urocortin increase anxiety-like behavior in the social interaction test via the CRF1 receptor Eur J Pharmacol 2005 509 145 153 10.1016/j.ejphar.2004.12.030 15733549

[B86] Moreau JL Kilpatrick G Jenck F Urocortin, a novel neuropeptide with anxiogenic-like properties Neuroreport 1997 8 1697 1701 10.1097/00001756-199705060-00027 9189917

[B87] Slawecki CJ Somes C Rivier JE Ehlers CL Neurophysiological effects of intracerebroventricular administration of urocortin Peptides 1999 20 211 218 10.1016/S0196-9781(98)00160-0 10422877

[B88] Sajdyk TJ Schober DA Gehlert DR Shekhar A Role of corticotropin-releasing factor and urocortin within the basolateral amygdala of rats in anxiety and panic responses Behav Brain Res 1999 100 207 215 10.1016/S0166-4328(98)00132-6 10212068

[B89] Venihaki M Sakihara S Subramanian S Dikkes P Weninger SC Liapakis G Graf T Majzoub JA Urocortin III, a brain neuropeptide of the corticotropin-releasing hormone family: modulation by stress and attenuation of some anxiety-like behaviours J Neuroendocrinol 2004 16 411 422 10.1111/j.1365-2826.2004.01170.x 15117334

[B90] Jamieson PM Li C Kukura C Vaughan J Vale W Urocortin 3 modulates the neuroendocrine stress response and is regulated in rat amygdala and hypothalamus by stress and glucocorticoids Endocrinology 2006 147 4578 4588 10.1210/en.2006-0545 16809443

[B91] Deussing JM Breu J Kuhne C Kallnik M Bunck M Glasl L Yen YC Schmidt MV Zurmuhlen R Vogl AM Urocortin 3 modulates social discrimination abilities via corticotropin-releasing hormone receptor type 2 J Neurosci 2010 30 9103 9116 20610744 10.1523/JNEUROSCI.1049-10.2010 PMC6632482

[B92] Lennartsson AK Jonsdottir IH Prolactin in response to acute psychosocial stress in healthy men and women Psychoneuroendocrinology 2011 36 1530 1539 10.1016/j.psyneuen.2011.04.007 21621331

[B93] Diaz-Moran S Palencia M Mont-Cardona C Canete T Blazquez G Martinez-Membrives E Lopez-Aumatell R Tobena A Fernandez-Teruel A Coping style and stress hormone responses in genetically heterogeneous rats: comparison with the Roman rat strains Behav Brain Res 2012 228 203 210 10.1016/j.bbr.2011.12.002 22178313

[B94] Torner L Neumann ID The brain prolactin system: involvement in stress response adaptations in lactation Stress 2002 5 249 257 10.1080/1025389021000048638 12475729

[B95] Pottinger TG Prunet P Pickering AD The effects of confinement stress on circulating prolactin levels in rainbow trout (Oncorhynchus mykiss) in fresh water Gen Comp Endocrinol 1992 88 454 460 10.1016/0016-6480(92)90240-K 1490590

[B96] Avella M Schreck CB Prunet P Plasma prolactin and cortisol concentrations of stressed coho salmon, Oncorhynchus kisutch, in fresh water or salt water Gen Comp Endocrinol 1991 81 21 27 10.1016/0016-6480(91)90121-L 2026313

[B97] Liu GX Cai GQ Cai YQ Sheng ZJ Jiang J Mei Z Wang ZG Guo L Fei J Reduced anxiety and depression-like behaviors in mice lacking GABA transporter subtype 1 Neuropsychopharmacology 2007 32 1531 1539 10.1038/sj.npp.1301281 17164814

[B98] Mennigen JA Martyniuk CJ Crump K Xiong H Zhao E Popesku J Anisman H Cossins AR Xia X Trudeau VL Effects of fluoxetine on the reproductive axis of female goldfish (Carassius auratus) Physiol Genomics 2008 35 273 282 10.1152/physiolgenomics.90263.2008 18765858

[B99] Benton CS Miller BH Skwerer S Suzuki O Schultz LE Cameron MD Marron JS Pletcher MT Wiltshire T Evaluating genetic markers and neurobiochemical analytes for fluoxetine response using a panel of mouse inbred strains Psychopharmacology (Berl) 2012 221 297 315 10.1007/s00213-011-2574-z 22113448 PMC3337404

[B100] Huang GJ Ben-David E Tort Piella A Edwards A Flint J Shifman S Neurogenomic evidence for a shared mechanism of the antidepressant effects of exercise and chronic fluoxetine in mice PLoS One 2012 7 e35901 10.1371/journal.pone.0035901 22558262 PMC3338479

[B101] Lee JH Ko E Kim YE Min JY Liu J Kim Y Shin M Hong M Bae H Gene expression profile analysis of genes in rat hippocampus from antidepressant treated rats using DNA microarray BMC Neurosci 2010 11 152 10.1186/1471-2202-11-152 21118505 PMC3009642

[B102] Griffith M Griffith OL Mwenifumbo J Goya R Morrissy AS Morin RD Corbett R Tang MJ Hou YC Pugh TJ Alternative expression analysis by RNA sequencing Nat Methods 2010 7 843 847 10.1038/nmeth.1503 20835245

[B103] Morey JS Ryan JC Van Dolah FM Microarray validation: factors influencing correlation between oligonucleotide microarrays and real-time PCR Biol Proced Online 2006 8 175 193 10.1251/bpo126 17242735 PMC1779618

[B104] Hawlena D Schmitz OJ Herbivore physiological response to predation risk and implications for ecosystem nutrient dynamics Proc Natl Acad Sci USA 2010 107 15503 15507 10.1073/pnas.1009300107 20713698 PMC2932623

[B105] Lima SL Anders Pape MÃ¸ller MM Peter JBS Stress and Decision Making under the Risk of Predation: Recent Developments from Behavioral, Reproductive, and Ecological Perspectives Advances in the Study of Behavior. Volume, Volume 27 1998 San Diego, CA USA: Academic Press Inc 215 290

[B106] Santos EM Kille P Workman VL Paull GC Tyler CR Sexually dimorphic gene expression in the brains of mature zebrafish Comp Biochem Physiol A Mol Integr Physiol 2008 149 314 324 10.1016/j.cbpa.2008.01.010 18289901

[B107] Toth AL Varala K Newman TC Miguez FE Hutchison SK Willoughby DA Simons JF Egholm M Hunt JH Hudson ME Robinson GE Wasp gene expression supports an evolutionary link between maternal behavior and eusociality Science 2007 318 441 444 10.1126/science.1146647 17901299

[B108] Sen Sarma M Whitfield CW Robinson GE Species differences in brain gene expression profiles associated with adult behavioral maturation in honey bees BMC Genomics 2007 8 202 10.1186/1471-2164-8-202 17603883 PMC1929079

[B109] Whitfield CW Cziko A-M Robinson GE Gene expression profiles in the brain predict behavior in individual honey bees Science (Washington D C) 2003 302 296 299 10.1126/science.1086807 14551438

[B110] Aubin-Horth N Landry CR Letcher BH Hofmann HA Alternative life histories shape brain gene expression profiles in males of the same population Proc Biol Sci 2005 272 1655 1662 10.1098/rspb.2005.3125 16087419 PMC1559854

[B111] Renn SC Aubin-Horth N Hofmann HA Fish and chips: functional genomics of social plasticity in an African cichlid fish J Exp Biol 2008 211 3041 3056 10.1242/jeb.018242 18775941 PMC3728697

[B112] Sanogo YO Hankison S Band M Obregon A Bell AM Brain transcriptomic response of threespine sticklebacks to cues of a predator Brain Behav Evol 2011 77 270 285 10.1159/000328221 21677424 PMC3182040

[B113] Catalan A Hutter S Parsch J Population and sex differences in Drosophila melanogaster brain gene expression BMC Genomics 2012 13 654 10.1186/1471-2164-13-654 23170910 PMC3527002

[B114] Drew RE Settles ML Churchill EJ Williams SM Balli S Robison BD Brain transcriptome variation among behaviorally distinct strains of zebrafish (Danio rerio) BMC Genomics 2012 13 323 10.1186/1471-2164-13-323 22817472 PMC3434030

[B115] Wong RY Ramsey ME Cummings ME Localizing brain regions associated with female mate preference behavior in a swordtail PLoS One 2012 7 e50355 10.1371/journal.pone.0050355 23209722 PMC3510203

[B116] Fitzpatrick MJ Ben-Shahar Y Smid HM Vet LEM Robinson GE Sokolowski MB Candidate genes for behavioural ecology Trends Ecol Evol 2005 20 96 104 10.1016/j.tree.2004.11.017 16701349

[B117] Wada K Howard JT McConnell P Whitney O Lints T Rivas MV Horita H Patterson MA White SA Scharff C A molecular neuroethological approach for identifying and characterizing a cascade of behaviorally regulated genes Proc Natl Acad Sci USA 2006 103 15212 15217 10.1073/pnas.0607098103 17018643 PMC1622802

[B118] Kanehisa M Goto S Sato Y Furumichi M Tanabe M KEGG for integration and interpretation of large-scale molecular data sets Nucleic Acids Res 2012 40 D109 D114 10.1093/nar/gkr988 22080510 PMC3245020

[B119] Kanehisa M Goto S KEGG: kyoto encyclopedia of genes and genomes Nucleic Acids Res 2000 28 27 30 10.1093/nar/28.1.27 10592173 PMC102409

[B120] Mennigen JA Lado WE Zamora JM Duarte-Guterman P Langlois VS Metcalfe CD Chang JP Moon TW Trudeau VL Waterborne fluoxetine disrupts the reproductive axis in sexually mature male goldfish, Carassius auratus Aquat Toxicol 2010 100 354 364 10.1016/j.aquatox.2010.08.016 20864192

[B121] Gunn BG Brown AR Lambert JJ Belelli D Neurosteroids and GABA(A) Receptor Interactions: A Focus on Stress Front Neurosci 2011 5 131 22164129 10.3389/fnins.2011.00131 PMC3230140

[B122] Zimmerberg B Brunelli SA Hofer MA Reduction of rat pup ultrasonic vocalizations by the neuroactive steroid allopregnanolone Pharmacol Biochem Behav 1994 47 735 738 10.1016/0091-3057(94)90181-3 7911579

[B123] Akwa Y Purdy RH Koob GF Britton KT The amygdala mediates the anxiolytic-like effect of the neurosteroid allopregnanolone in rat Behav Brain Res 1999 106 119 125 10.1016/S0166-4328(99)00101-1 10595427

[B124] Evans J Sun Y McGregor A Connor B Allopregnanolone regulates neurogenesis and depressive/anxiety-like behaviour in a social isolation rodent model of chronic stress Neuropharmacology 2012 63 1315 1326 10.1016/j.neuropharm.2012.08.012 22939998

[B125] Engin E Treit D The anxiolytic-like effects of allopregnanolone vary as a function of intracerebral microinfusion site: the amygdala, medial prefrontal cortex, or hippocampus Behav Pharmacol 2007 18 461 470 10.1097/FBP.0b013e3282d28f6f 17762514

[B126] Homberg JR Olivier JD Blom T Arentsen T van Brunschot C Schipper P Korte-Bouws G van Luijtelaar G Reneman L Fluoxetine exerts age-dependent effects on behavior and amygdala neuroplasticity in the rat PLoS One 2011 6 e16646 10.1371/journal.pone.0016646 21304948 PMC3031607

[B127] Bouet V Klomp A Freret T Wylezinska-Arridge M Lopez-Tremoleda J Dauphin F Boulouard M Booij J Gsell W Reneman L Age-dependent effects of chronic fluoxetine treatment on the serotonergic system one week following treatment Psychopharmacology (Berl) 2012 221 329 339 10.1007/s00213-011-2580-1 22205158

[B128] Olivier JD Blom T Arentsen T Homberg JR The age-dependent effects of selective serotonin reuptake inhibitors in humans and rodents: A review Prog Neuropsychopharmacol Biol Psychiatry 2011 35 1400 1408 10.1016/j.pnpbp.2010.09.013 20883714

