# Supplementary figures and images for: Behavioral and neurogenomic transcriptome changes in wild-derived zebrafish with fluoxetine treatment
Source: BMC Genomics. 2013 May 24;14:348. doi: 10.1186/1471-2164-14-348 (PMC3667115; doi:10.1186/1471-2164-14-348)

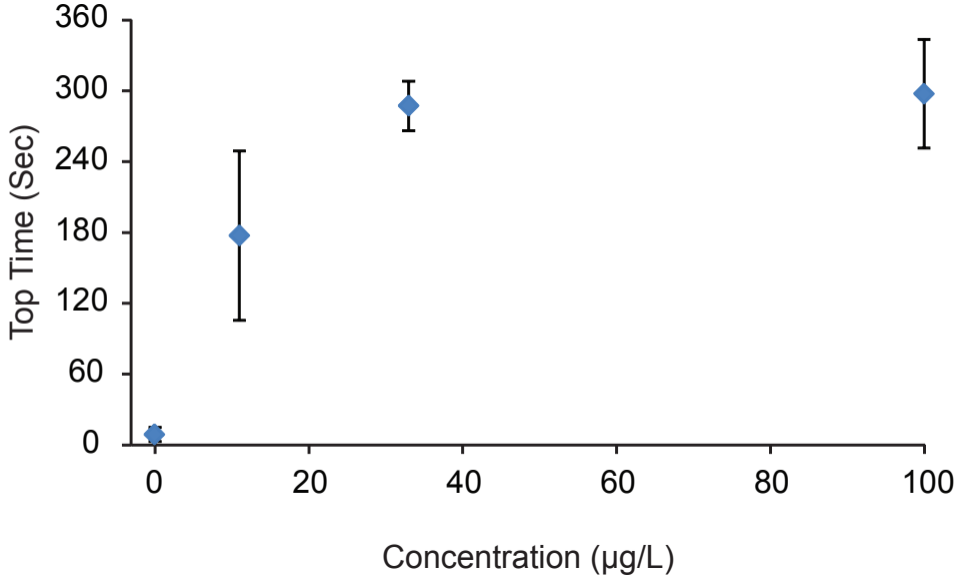

Supplement: Additional file 1: Figure S1 — Fluoxetine dose-response curve. Time spent on the top half of the tank (y-axis) for fish treated with 0, 11, 33, and 100 μg/L racemic fluoxetine (x-axis). N = 6 at each concentration. [file 1471-2164-14-348-S1.pdf]

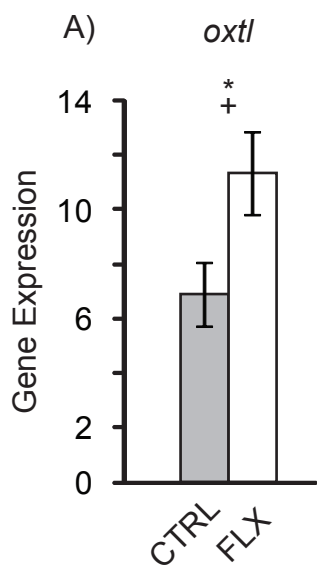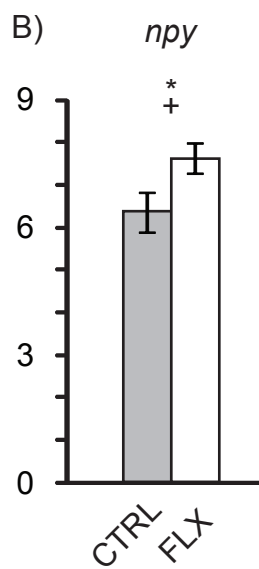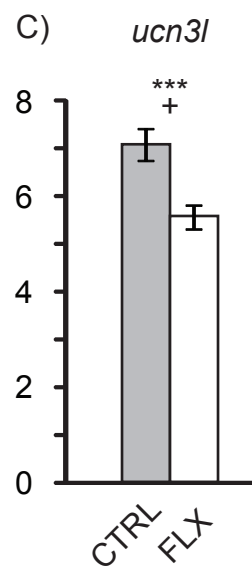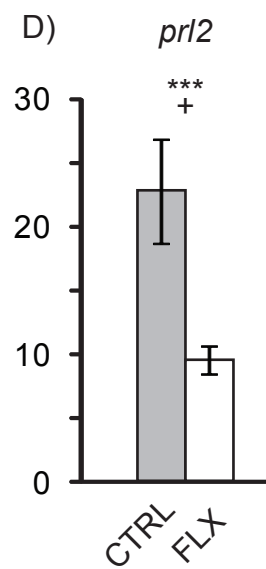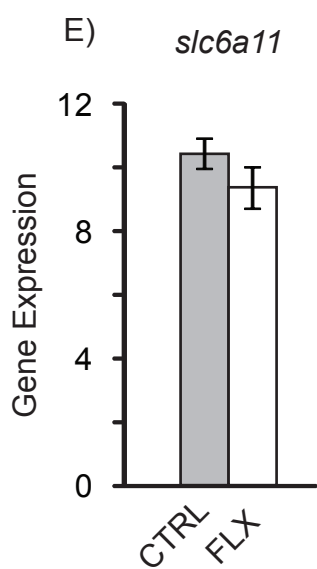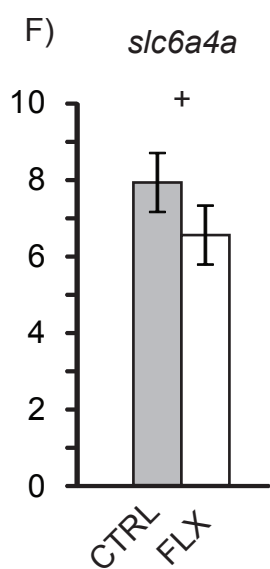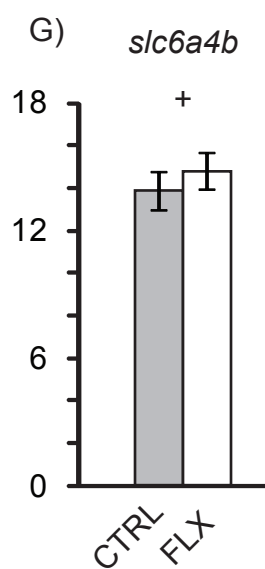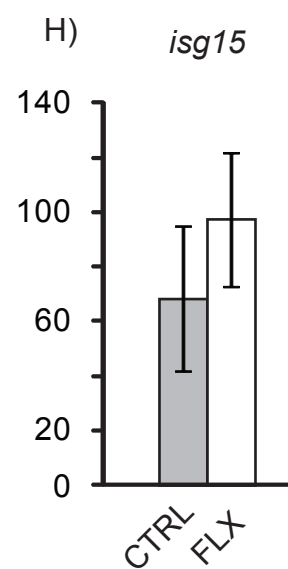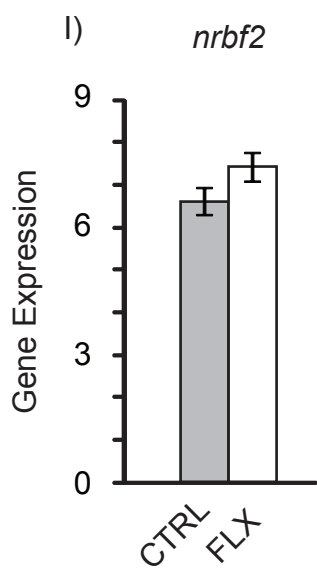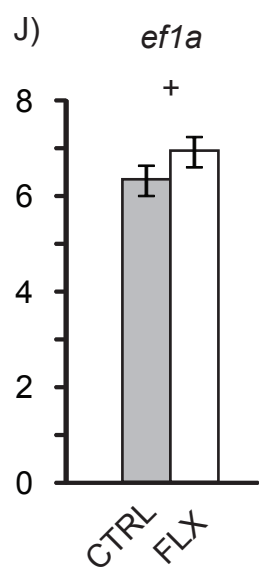

Supplement: Additional file 3: Figure S2 — qRT-PCR validation of expression of select genes with racemic fluoxetine treatment. Genes examined are (A) isotocin, (B) neuropeptide Y, (C) urocortin 3, (D), prolactin, (E) GABA transporter, (F) serotonin transporter A, (G) serotonin transporter B, (H) ISG15 ubiquitin-like modifier, (I) nuclear receptor binding factor 2, and (J) elongation factor 1-alpha. Gene expression values are normalized to total RNA input. Error bars represent standard error. *, p < 0.05; **; ***, p < 0.001; +, data is consistent with RNA-sequencing results. [file 1471-2164-14-348-S3.pdf]
