# Supplementary material for: Behavioral and neurogenomic transcriptome changes in wild-derived zebrafish with fluoxetine treatment
Source: BMC Genomics. 2013 May 24;14:348. doi: 10.1186/1471-2164-14-348 (PMC3667115; doi:10.1186/1471-2164-14-348)
Supplement: Additional file 2: Table S1 — qRT-PCR primer characteristics. [file 1471-2164-14-348-S2.pdf]

Supplementary Table 1. qRT-PCR primer characteristics

Gene Symbol	Forward Primer	Reverse Primer	Amplicon Length
SLC6A11	5’-ACCCATATTCCACATCACCAG-3’	5’-CTATAGCGAGACAAAGGGCG-3’	117
SLC6A4B	5’-GCCGAGGAGTTTTACACGAGGA-3’	5’-ACATATGGCAGGGTGGCAGT-3’	188
NRBF2	5’-GTGTGGTTGAGTCTGTAGTCG-3’	5’-AGCTTTTCTGTGGCAGGAG-3’	140
PRL2	5’-CTCTCCAATGACCTGCACTC-3’	5’-CTCCTCGCGAGCAAGTTTC-3’	144
OXTL	5’-ATTCGACAGTGTATGCCGTG-3’	5’-TCACACGGAGAAGGGAGAAA-3’	146
NPY	5’-ACTACATCAACCTCATAACAAGGC-3’	5’-GATGAGATCACCATGCCAAATGAT-3’	138
EF1A	5’-CCTCTTGGTCGCTTTGC-3’	5’-GGTGTGATTGAGGGAAATTCA-3’	150
UCN3L	5’-TGATAGTGACTCGCTTGCCT-3’	5’-CATTGTTCTGCCCTGCACTC-3’	123
SLC6A4A	5’-TAACCACTACAGTTTGGCTTGATG-3’	5’-AACAGTTAACCGAGCTTGTGAT-3’	147
ISG15	5’-TCATAACTCGGTGACGATGCAG-3’	5’-TGATCCGCTGACCGTTTTCG-3’	170

qRT-PCR parameters (all genes): 2 min at 50°C, 2 min at 95°C, followed by 40 cycles at 15 sec 95°C and 1 min 60°C. Primer concentration was 5 pmol for all genes except SLC6A4A (1.5 pmol).
